# Gut Microbiota-Derived Short-Chain Fatty Acids: Impact on Cancer Treatment Response and Toxicities

**DOI:** 10.3390/microorganisms10102048

**Published:** 2022-10-17

**Authors:** Ghanyah H. Al-Qadami, Kate R. Secombe, Courtney B. Subramaniam, Hannah R. Wardill, Joanne M. Bowen

**Affiliations:** 1 School of Biomedicine, University of Adelaide, Adelaide, SA 5005, Australia; 2 The University of Queensland Diamantina Institute, The University of Queensland, Woolloongabba, QLD 4102, Australia; 3 Supportive Oncology Research Group, Precision Medicine (Cancer), South Australian Health and Medical Research Institute, Adelaide, SA 5000, Australia

**Keywords:** microbiota, short-chain fatty acids, cancer, chemotherapy, radiotherapy, immunotherapy, treatment response, supportive care

## Abstract

The gut microbiota has emerged as a key modulator of cancer treatment responses in terms of both efficacy and toxicity. This effect is clearly mediated by processes impacting the activation and modulation of immune responses. More recently, the ability to regulate chemotherapeutic drug metabolism has also emerged as a key driver of response, although the direct mechanisms have yet to be fully elucidated. Through fermentation, the gut microbiota can produce several types of metabolites, including short-chain fatty acids (SCFAs). SCFAs play an important role in maintaining epithelial barrier functions and intestinal homeostasis, with recent work suggesting that SCFAs can modulate response to cancer treatments and influence both anti-tumor immune response and inflammatory-related side effects. In this review, we will discuss the importance of SCFAs and their implications for cancer treatment response and toxicities.

## 1. Introduction

The collection of bacteria and other microorganisms residing in the gastrointestinal tract, termed the gut microbiota, has emerged as an important target in improving and personalizing oncology treatment. A growing body of research has shown potential roles for specific microbial taxa in the efficacy of cancer treatment, as well as in the development of associated toxicities [1,2,3]. The richness of the microbiome has also been investigated, with higher microbial diversity shown to be a key predictor of survival in people having chemoradiation for cervical cancer [4]. A seminal study by Gopalakrishnan et al. [1] clearly showed significant differences in both the diversity and composition of the gut microbiota of people who responded to PD-1 inhibitor immunotherapy for melanoma compared to non-responders. These compositional differences may lead to altered treatment response in a variety of ways, including via changes in direct drug metabolism (for example the action of beta-glucuronidase in the intestinal toxicity of irinotecan [5]), or modulation of the host immune responses [6,7].

With the advent of more sophisticated analytical methods, there is now more appreciation for the functional aspects of the microbiota, including the production of metabolites. Short-chain fatty acids (SCFAs) are one important class of metabolites produced by the gut microbiota. SCFAs, which include butyrate, acetate, propionate, and others (discussed below), are produced in the gut by bacterial fermentation of indigestible fibers and have a variety of functions both in the gut and distal sites including the brain and kidneys [8]. SCFAs have previously been linked to colorectal cancer development, with multiple studies showing that butyrate, propionate, and acetate induce apoptosis in colorectal cancer cells but not in healthy cells. In addition, some studies have suggested that butyrate and propionate have potent anti-neoplastic effects [9]. This has led to suggestions that SCFA manipulation may be a useful preventative or therapeutic strategy in a variety of cancers [9,10,11,12,13]. 

Results linking the microbiota and cancer treatment outcomes have been promising but have so far failed to provide significant improvements to clinical practice [14]. It is therefore now increasingly important to move beyond simply correlating the abundance of particular bacterial taxa with a disease or phenotype, and to understand how the functional capacity of the microbiota may be critical [15]. Furthermore, in the development of microbial-based therapeutics, there is a strong rationale to investigate metabolites such as SCFAs, rather than bacteria themselves. This is because direct metabolite supplementation would remove the need to ensure microorganisms successfully colonize the host, such is the case with probiotics. This review will examine current literature and suggest future research paths to understand the possible benefits and uses of SCFAs in optimizing cancer treatment and response.

A semi-structured search of PubMed for full-text articles in English was completed using search terms including short chain fatty acid, cancer treatment, cancer treatment toxicities, mucositis, graft-versus host disease, chemotherapy, radiotherapy and immunotherapy. The findings of the key studies identified were summarised in Table 1 and Table 2.

## 2. Short-Chain Fatty Acids

**Figure 1:**
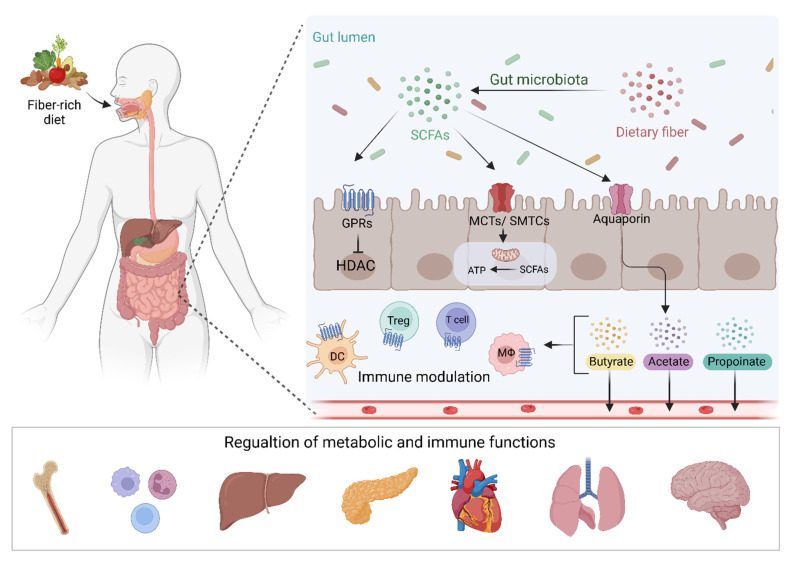
Dietary fiber and other fermentable substrates are fermented by the gut microbiota leading to the production of SCFAs. These metabolites interact with GPCRs or MCTs and SMTCs transporters leading to the regulation of gene transcription and energy productions in IECs. SCFAs can also passively cross the intestinal mucosa and regulate intestinal immunity and pass into the circulation to modulate metabolic and immune functions in different body organs. SCFAs; short-chain fatty acids, GPRs; G protein-coupled receptors, MCTs; Monocarboxylate transporters, SMTCs; Sodium-coupled monocarboxylate transporter; DC, dendritic cell, Treg; T regulatory cell, Mϕ; Macrophage.

SCFAs are small organic carboxylic acids with 1 to 6 carbon atoms, and in the intestine, are the main product of anaerobic fermentation of indigestible polysaccharides such as resistant starch, inulin, cellulose, and pectin (Figure 1) [16]. Acetate, propionate, and butyrate are the most commonly produced SCFAs in the human gut, in a roughly 3:1:1 ratio [17]. Other SCFAs include formate, isobutyrate, valerate, isovalerate, and 2-methylbutanoate. SCFAs can move from the gut via the bloodstream in differing amounts. A study using stable isotopes in healthy human subjects found that systemic availability of acetate, propionate, and butyrate was 36%, 9%, and 2%, respectively, [18]. Subsequently, SCFAs have a range of functions both in the gut and elsewhere, with differences in local and systemic effects governed by their systemic availability. These include being a key energy source for colonocytes and playing roles in G-protein coupled receptor (GPCR) binding, histone deacetylation, and immune modulation [16,19]. SCFA quantification in fecal samples is commonly used to quantify SCFA production. This may however not be an accurate measure, as previous research has suggested that a majority of SCFAs produced in the colon are absorbed by the gut mucosa [16,20]. In addition, systemic SCFA levels may be affected by gut epithelial integrity [21].

A wide range of bacteria, many of which have previously been implicated in the efficacy or toxicity of cancer treatment, can produce SCFA, with the amounts and types of SCFA produced dependent on the types of bacteria present. Acetate is the SCFA produced in the highest levels in the gut. This is due to acetate production pathways being widely distributed among multiple types of bacteria, whereas other SCFAs such as butyrate and propionate production is restricted to a small group of bacterial types [22]. The phylum Firmicutes is the main butyrate-producing taxa, particularly *Faecalibacterium prausnitzii (F. prausnitzii)* (Ruminococcaceae) and *Roseburia* spp. (Lachnospiraceae). *Eubacterium* and *Coprococcus* are also important butyrate-producers [23]. These key butyrate-producing bacteria are generally anaerobes, and therefore flourish in the low-oxygen environment of the colon. In addition, *Bifidobacterium* species are also able to produce acetate and lactate, and *Akkermansia muciniphila,* among others, produce propionate and acetate, with most propionate producers in the colon belonging to the Bacteroidota phylum [8,16,24]. Luminally, SCFAs serve to acidify their environment, and in doing so restrict the growth of pathogenic microbes by preventing cellular respiration [25]. Similarly, SCFAs such as acetate are potent nutrient sources for other commensal microbes (including butyrate producers), and this cross-feeding mechanism is critical for the maintenance of a healthy and diverse microbial ecosystem.

SCFAs are absorbed by colonocytes via hydrogen or sodium-dependent monocarboxylate transporters [19], where much is used as an energy source for these cells. SCFAs, primarily butyrate and propionate, not metabolized within colonocytes are transported into the portal circulation and often then used as an energy source for hepatocytes [26,27]. Butyrate is a particularly important energy source for colonocytes, with previous research showing that colonizing germ-free mice with butyrate-producing bacteria increased oxidative phosphorylation and contained autophagy to normal levels in the gut [28]. Butyrate is also important in stabilizing gut epithelial barrier function, via the consumption of local oxygen molecules and subsequent stabilization of the barrier protecting transcription factor hypoxia-inducible factor (HIF) [29]. 

Aside from the above-mentioned monocarboxylate transporters, SCFAs can activate G protein-coupled receptors (GPCR), also known as free fatty acid receptors [16,30]. GPR41, GRP43, and GPR109A can be activated by multiple SCFAs and subsequently inhibit the production of cAMP [31]. These GPCRs are expressed on epithelial cells, neutrophils, and macrophages both within and outside of the gut, as well as a variety of other cell types including sympathetic neurons and pancreatic beta cells [32]. The wide range of GPCR sites shows the broad potential for SCFA-mediated effects. Enteroendocrine cells also express these receptors and respond by releasing contents that regulate neuroendocrine pathways controlling local and distant activity. Activities include appetite regulation, glucose homeostasis, and mucosal growth. See van der Hee and Wells [32] for further discussion of these effects. 

Butyrate and propionate can modulate inflammatory responses in the gut mucosa via histone deacetylase (HDAC) inhibition. Previous research has shown that butyrate can suppress colonic inflammation via HDAC1-dependent Fas upregulation [33]. SCFAs can also alter the immune response in numerous other ways [34]. For example, butyrate can induce Treg differentiation [35] and increase the generation of Th1 and Th17 cells [36]. Microbiota-derived SCFA has also been shown to promote the cellular metabolism of antigen-activated CD8^+^ T cells and enhance their differentiation into long-term memory T cells [37]. Combined, these functions support the central role of SCFAs in the modulation of the immune system. 

While it is beyond the scope of this paper to describe all individual differences in metabolism and function of different SCFAs, it is important to note that each SCFA has specific functions, which may be important in understanding their role in cancer treatment outcomes. As mentioned above, pathways for acetate production are spread amongst a range of bacterial types, leading to its high production in the gut, of which a relatively high level is able to move into the bloodstream. Acetate also has specific functions systemically, including involvement in lipid synthesis and acetylation reaction [38]. Butyrate is primarily produced via butyryl CoA:acetate CoA transferase pathways, and is one of the most researched SCFAs, due to its important role as an energy source. Propionate is produced via two pathways; the succinate pathways and the propanediol pathway. See Deleu, Machiels [8] for further description of SCFA production pathways. Propionate has also been suggested to have roles in lipogenesis in hepatocytes, as well as having antiproliferative effects in colon cancer cell lines [39]. 

## 3. SCFAs and Cancer Treatment Response

**Figure 2:**
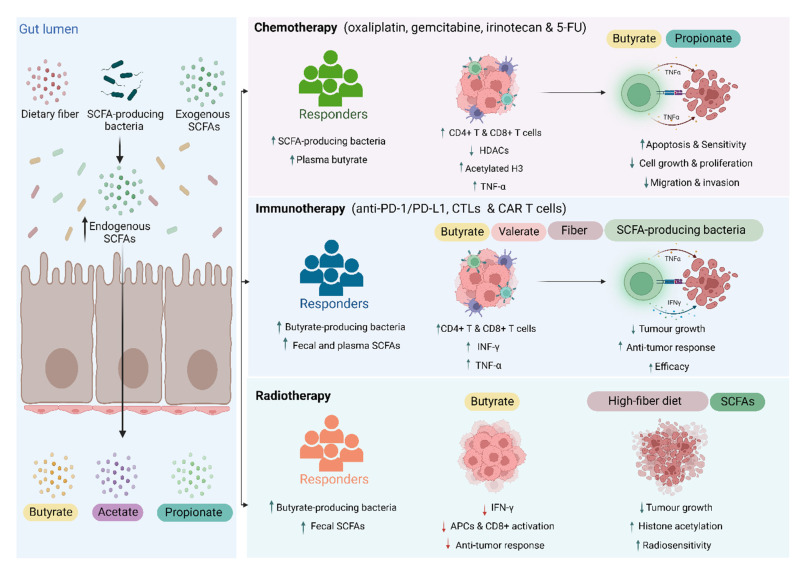
Role of SCFAs in cancer treatment based on current evidence. For chemotherapy, responders to treatment had a higher abundance of SCFA-producing microbiota and higher levels of plasma butyrate. Butyrate and propionate can increase the intratumoral T cells and TNF-α and reduce HDACs and hence increase chemosensitivity, tumor apoptosis and inhibit cell growth, migration, and invasion. Responders to immunotherapy also had a higher abundance of butyrate-producing microbes and higher levels of fecal and plasma SCFAs. SCFAs (butyrate and valerate), fiber, or SCFA-producing bacteria supplements increase the intratumoral T cells, INF-γ and TNF-α and result in inhibition of tumor growth and improving anti-tumor immune response. For radiotherapy, a higher abundance of butyrate-producing bacteria and higher levels of fecal SCFAs correlates to a better response. While butyrate administration was found to reduce anti-tumor efficacy, the administration of total SCFAs or a fiber-rich diet improved tumor radiosensitivity. SCFAs, short chain fatty acids; 5-FU, 5-fluorouracil; HDACs, histone deacetylases; TNF-α, tumor necrosis factor-alpha; PD-1, programmed cell death protein 1; PD-L1, programmed death-ligand 1; CTLs, cytotoxic T cells; CAR T cells, chimeric antigen receptor T cells; INF-γ, interferon-gamma; APCs, antigen-presenting cells.

A body of evidence is developing that SCFAs may have a role in the efficacy of various cancer treatment types, including chemotherapy, immunotherapy, and radiotherapy. Key to this is the immunomodulatory properties of SCFA that can alter anti-tumor effects, such as amounts of tumor-killing CD4+ and CD8+ T cells, as well as immune-suppressing Tregs [40]. Additionally, HDACs, of which butyrate in particular can inhibit, are linked to cell cycle regulation and proliferation, with a variety of other HDAC inhibitors tested as anti-cancer agents [41]. Different treatment modalities affect the gut microbiota differently and therefore may interact with SCFA in different ways (Figure 2).

### 3.1. Chemotherapy 

Accumulating data suggests that SCFAs, particularly butyrate and propionate, could impact the efficacy of chemotherapy by enhancing tumor sensitivity to chemotherapeutic agents or augmenting anti-tumor immune responses (Table 1). It has been shown that decreased abundance of SCFA-producing taxa (*Coprococcus*, *Dorea,* and uncultured *Ruminococcus*) is linked to lower efficacy of neoadjuvant chemotherapy (cyclophosphamide, anthracycline, taxol, or herceptin) in patients with breast cancer, and subsequently associated with a lower number of intratumoral CD4^+^ and CD8^+^ cells and peripheral CD4+ T cells [42] (Figure 2). More specifically, He et al. demonstrated that, in a mouse model, butyrate enhances the efficacy of oxaliplatin by enhancing the anti-tumor activity of intratumoral and draining lymph node CD8+ T cells in an IL-12 signaling pathway-dependent manner. In the same study, the authors found that oxaliplatin responder patients with gastrointestinal cancer had higher levels of plasma butyrate compared to non-responders [43].

**Table 1:** Impact of SCFA-based interventions on cancer treatment anti-tumor efficacy.

Ref.	Subjects/Model	Treatment	Key Findings
Geng et al. [44]	HCT116 human colorectal cancer cell line	5-FU +/− butyrate	Butyrate enhanced 5-FU-induced apoptosis on colorectal cancer cells Butyrate improved cell sensitivity to 5-FU by augmenting 5-FU-induced inhibition of DNA synthesis.
Encarnação et al. [45]	WiDr, C2BBe1, and LS1034 colorectal cancer cells WiDr colorectal mouse model	Irinotecan +/− butyrate	In vitro, butyrate reduced the IC50 of irinotecan by enhancing cancer cell apoptosis and reducing proliferation. Butyrate significantly decreased the expression of chemoresistant-related protein. In vivo, butyrate delayed tumor growth following irinotecan treatment.
Panebianco et al. [46]	BxPC-3 and PANC-1 pancreatic cancer cell line BxPC-3 pancreatic cancer mouse model	Gemcitabine +/− butyrate	In vitro, butyrate administration enhanced gemcitabine-induced inhibition of cancer cell growth and apoptosis. In vivo, butyrate did not affect tumor volume but suppressed stromatogenesis by reducing the density of stroma collagen bundles, and expression of myofibroblasts, vascular architecture, and M2-polarized macrophage markers in tumors.
Li et al. [47]	HGC-27 and SGC-7901 gastric cancer cell lines SGC-7901 gastric cancer mouse model	Cisplatin +/− butyrate	Butyrate synergized cisplatin-induced tumor cell apoptosis by increasing mitochondrial ROS levels and mitochondrial membrane potential. Suppressed cell migration and invasion in vitro by reducing the levels of MMP-2, -9 proteins. Cisplatin augmented cisplatin-induced suppression of tumor growth by increasing the levels of apoptosis makers.
Kobayashi et al. [48]	HepG2, HuH-7, JHH-4 hepatocellular carcinoma cell lines HepG2 human hepatocellular carcinoma mouse model	Cisplatin +/− propionate	In vitro, combined therapy inhibited proliferation and enhanced apoptosis through the GPR41 signaling pathway. Enhanced levels of activated DNA fragmentation markers (cleaved caspase-3). Enhanced the expression of TNF-α by downregulating the expression of HDACs and enhancing histone H3 acetylation. In vivo, combined therapy suppressed tumor growth and enhanced histone H3 acetylation and mRNA expression of TNF-α.
He et al. [43]	MC38 colon cancer/ EG7 lymphoma mouse model Patients with gastrointestinal cancer (*n*= 21)	Oxaliplatin +/− butyrate	Butyrate augmented oxaliplatin efficacy by enhancing the anti-tumor activity of CD8+ T cells in an IL-12 signaling pathway-dependent manner. Butyrate prompted cytotoxic CD8+ T cell anti-tumor responses in vitro and in vivo through the IL-12 signaling pathway. Clinically, responders to oxaliplatin had higher concentrations of serum butyrate compared to non-responders.
Zhang et al. [49]	MC38 colon cancer mouse model	Anti-PD-1 +/− butyrate	Pre- immunotherapy butyrate supplement improved anti-PD-1 efficacy in mice humanized with gut microbiota from CRC patients Butyrate increased infiltration of tumor-killing CD4+ and CD8+ cells in the tumor.
Luu et al. [10]	B16-OVA melanoma/ PancOVA pancreatic cancer mouse model	CTLs and CAR T cells +/− valerate / butyrate	Transferring valerate or butyrate-treated cytotoxic T cells and chimeric antigen receptor T cells into tumor-bearing mice increased the production of CD25, IFN-γ, and TNF-α and enhances the anti-tumor activity.
Jing et al. [50]	MC38 colon adenocarcinoma mouse model	Anti-PD-L1 +/− fiber-rich powder or SCFAs	Administrating fiber-rich powder improved anti-PD-L1 efficacy by increasing the production of acetate, propionate, butyrate, and valerianate. Oral butyrate supplements did not affect anti-PD-L1 efficiency or total leukocytes and CD8+ T cell proportion. Oral SCFAs did not increase cecum or colon SCFA levels In vitro, culturing lymphocytes with SCFAs increased the proportion of CD8+ T cells.
Han et al. [51]	CT26 colon cancer mouse model	Anti-PD-1 +/− Inulin (dietary fiber) or SCFAs	Inulin improved anti-tumor response and delayed tumor growth by increasing systemic tumor-specific CD8+ cells count. Inulin increased SCFA-producing *Lactobacillus, Akkermansia,* and *Roseburia,* and fecal SCFAs. Negative correlation between the tumor size and the fecal propionate and butyrate. Inulin increased systemic tumor-specific CD8+ cells, splenic IFN-γ+ CD8+ T cells, intratumoral CD8+, CD4+, activated dendritic cells and decreased PD-1 positive CD8+ cells. Oral administration of free SCFAs did not improve the anti-tumor response. Antibiotic treatment and GPR43 knockdown abrogated anti-tumor activity. In vitro, SCFAs enhanced the memory response of IFN-γ+ CD8+ T cells and upregulated T-cell factor 1.
Spencer et al. [52]	Patients with metastatic melanoma (*n* = 128) B2905 and HMel melanoma mouse model	Anti-PD-1 +/− fiber-rich diet	High fiber dietary intake improved progression-free survival in patients. Fiber-rich diet delayed tumor growth in mice. Fiber-rich diet had no impact on tumor response in germ-free mice. Higher propionate levels were observed in mice receiving a fiber-rich diet. Fiber-rich diet increased the number of tumor-infiltrating CD4+ cells and IFNγ+ cytotoxic T cells.
Coutzac et al. [12]	Patients with metastatic melanoma (*n* = 85) CT26 and MC38 Colon/ MCA101_OVA_ fibrosarcoma mouse model	Ipilimumab +/− systemic butyrate	Higher serum levels of propionate and butyrate were associated with poor clinical outcome and high serum SCFAs was positively correlated to the proportion of Tregs. Butyrate administration reduced treatment efficacy by suppressing dendritic cell maturation and decreased T-cell expansion and functions in mice.
Tomita et al. [53,54]	Patients with advanced non–small cell lung cancer received antibiotics or PPI (*n* = 118)	Immune checkpoint blockade +/− *C. butyricum*	*C. butyricum* was associated with longer progression-free and overall survival, particularly in patients who received PPI with/without antibiotics. *C. butyricum* administration increased beneficial microbiota and reduced oral-related pathobionts in the gut.
Then et al. [55]	RT112 bladder carcinoma mouse model RT112 bladder cell line	Radiation+ fiber-containing diet Cells irradiation +/− SCFAs	Soluble high-fiber diet delayed tumor growth following irradiation Soluble high-fiber diet increased acetate-producing *Bacteroides acidifaciens,* which was associated with better radiation response and long survival. In vitro, SCFAs increased histone acetylation and reduced cell proliferation while butyrate only significantly enhanced radiosensitivity.
Yang et al. [56]	MC38 colon/ B16F1 melanoma mouse model	Local radiation +/− Systemic or intratumoral butyrate	Both systemic and intratumoral butyrate impaired anti-tumor response in MC38 and B16F1 models. Intratumoral butyrate did not directly protect tumor cells from radiation but inhibited radiation-induced anti-tumor immune responses. Intratumoral butyrate inhibited type I IFN expression in dendritic cells and hence suppressed dendritic cell functions and activation of CD8^+^ T cell immune responses.
Uribe-Herranz et al. [57]	B16OVA melanoma/ TC-1 lung cancer mouse model	Local irradiation +/− butyrate	Depletion of butyrate-producing taxa and reducing tumor butyrate levels by vancomycin improved anti-tumor activity. Butyrate administration reduces antigen-presenting cells activation and functions.

Butyrate has also been found to enhance the activity of 5-fluorouracil (5-FU) in colon cancer cells [44]. Furthermore, treating pancreatic carcinoma cell lines with sodium butyrate increases cell sensitivity to SN-38, cisplatin, and fluorouracil chemotherapies by inducing histone acetylation and p53 expression and subsequently increasing apoptosis [58]. A similar synergic effect was also observed after combined butyrate treatment with docetaxel, irinotecan, gemcitabine, or cisplatin both in vitro and in vivo against various types of tumors [45,46,47,59]. The co-administration of docetaxel and butyrate enhanced docetaxel response by upregulation and downregulation of apoptosis and proliferation-related proteins, respectively, [59]. Further, butyrate reduced irinotecan’s half maximal inhibitory concentration, expression of the chemoresistant-related protein P-glycoprotein and enhanced cancer cell apoptosis and anti-tumor efficiency of irinotecan against colon cancer cell lines [45]. Combined gemcitabine and butyrate treatment also further prompted apoptosis and reduced tumor-associated stromatogenesis in pancreatic ductal adenocarcinoma models [46]. Additionally, butyrate enhanced tumor apoptosis and the suppression of tumor growth, migration, and invasion capacity of gastric cancer cell lines treated with cisplatin [47] (Figure 2). Similar to butyrate, it has been demonstrated that propionate enhances the anti-tumor activity of cisplatin against human hepatocellular carcinoma cells. This was mediated by a GPR41-dependent reduction in the expression of HDACs and hence the enhancement of TNF-α expression [48] (Figure 2). Overall, evidence suggests that increased levels of SCFAs lead to increased immune cell killing of tumor cells, and less tumor cell growth. 

### 3.2. Immunotherapy 

Due to the critical role SCFAs play in regulating immune responses, as well as the importance of microbiota composition in immunotherapy, SCFAs potential ability to augment immunotherapy efficacy is receiving increasing attention. While some studies have reported that SCFAs negatively impact the immunotherapy response, the majority of the current evidence supports a positive role (Table 1). It has been widely shown that responders to immunotherapy, such as ipilimumab and nivolumab, have an increased abundance of butyrate-producing bacteria compared to non-responders [1,3,60] (Figure 2). For instance, *F. prausnitzii* is a butyrate-producing bacteria that have been associated with better treatment response and prolonged progression-free survival in patients treated with immunotherapy [1]. Further, the administration of butyrate-producing *Clostridium butyricum* (*C. butyricum*) was found to improve both progression-free and overall survival in patients with lung cancer treated with immune checkpoint inhibitors [53,54]. Additionally, Botticelli et al. analyzed the metabolomic profile of 11 patients with non-small cell lung cancer treated with nivolumab immunotherapy and demonstrated that higher levels of fecal SCFAs including butyrate and propionate were associated with long responders (>1-year progression-free survival) [61]. Similarly, Nomura et al. analyzed SCFAs from 52 patients with solid tumors treated with immunotherapy (nivolumab or pembrolizumab) and reported that higher fecal SCFA concentration (acetic, propionic, butyric, valeric acid) and plasma isovaleric acid prior to treatment initiation was found in responders compared to non-responders and was associated with longer progression-free survival [11]. Mechanistically, Zhang et al. found that butyrate enhanced the efficacy of an anti-PD-1 by promoting the infiltration of T cells in the tumor microenvironment in mice humanized with gut microbiota obtained from patients with colorectal cancer (CRC) [49]. Further, in mouse models with melanoma or pancreatic tumors, Luu et al. reported that microbial-derived valerate and butyrate improved the anti-tumor activity of cytotoxic T cells and chimeric antigen receptor (CAR) T cells. A mechanistic in vitro study in this paper showed that treatment of these cells with the SCFAs valerate and butyrate enhanced the production of CD25, IFN-γ, and TNF-α by both cytotoxic T cells and CAR T cells [10] (Figure 2). A range of other in vivo studies have also shown similar results, with high-fiber diets, and subsequently increased SCFA levels, improving response to various immunotherapies [50,51,52].

Conversely, some evidence has suggested a negative role of SCFAs in ipilimumab efficacy. Coutzac et al. investigated the association between SCFAs and response to ipilimumab in patients with melanoma and a melanoma mouse model [12]. They first assessed the gut microbiome of 88 patients with metastatic melanoma treated with ipilimumab and reported that higher proportions of *F. prausnitzii* were associated with prolonged progression-free and overall survival. However, serum SCFA measurements showed that *F. prausnitzii* was negatively correlated to serum butyrate. Butyrate concentration was also negatively correlated to progression-free and overall survival. High butyrate and propionate in the blood were associated with resistance to CTLA-4 blockade and a higher proportion of Treg cells. The negative correlation between the abundance of butyrate-producing *F. prausnitzii* and serum butyrate levels could be explained by the increase in systemic butyrate levels due to the disruption of intestinal barrier integrity caused by ipilimumab. Elevated levels of serum SCFAs have been reported previously in patients with type 2 diabetes and this was also associated with higher levels of serum zonula occludens-1, an indicator of a leaky gut [62]. However, the negative impact of butyrate on anti-tumor immunity is not explained. In the same abovementioned ipilimumab study, authors found that in a mouse model, butyrate administration impaired anti-tumor immune response by impacting dendritic cell maturation and T cell function [12]. Another mouse study also showed that oral butyrate supplementation did not enhance anti-PD-L1 treatment, although did not decrease efficacy either [50]. These results may suggest that the efficacy of immunotherapy is not universally enhanced by increased SCFAs and may be population-dependent. Alternatively, measurement of serum SCFA, which depends on the production of SCFA as well as absorption in the gut and elsewhere in the body, may lead to different conclusions than studies only measuring fecal SCFA.

### 3.3. Radiotherapy 

Currently, there is contradicting evidence on the role of SCFA in radiotherapy efficacy (Table 1). Sánchez-Alcoholado and colleagues showed that, in patients with colorectal cancer treated with neoadjuvant radiochemotherapy, the gut microbiota of responders was enriched with butyrate-producing bacteria, compared to treatment non-responders. These patients also had significantly higher fecal levels of SCFAs (acetate, butyrate, and hexanoic and isobutyric acids) compared to non-responders [63]. Further, Yi et al. reported an increase in butyrate-producing bacteria (e.g., *Roseburia, Dorea*, and *Anaerostipes*) were enriched in responders to chemoradiotherapy in patients with locally advanced rectal cancer [64] (Figure 2). In contrast, pre-clinical studies have found that butyrate administration impairs the anti-tumor response of ionizing radiation [56,57]. Yang et al. administered germ-free mice with *Kineothrix alysoides*, a member of the butyrate-producing *Lachnospiraceae* family. This resulted in decreased ionizing radiation efficacy, with increased systemic and tumor levels of butyrate. As butyrate can suppress the expression of type I interferons in dendritic cells and the cross-presentation of tumor-associated antigens to tumor-specific cytotoxic T cells; it was suggested that this mechanism led to reduced anti-tumor immune response [56] (Figure 2). 

Other studies have used indirect methods of altering SCFA output, with varying results. Uribe-Herranz et al. demonstrated that treating tumor-bearing mice with the antibiotic vancomycin improved radiation-induced anti-tumor activity. Butyrate-producing taxa were depleted by vancomycin and subsequently reduced butyrate levels in tumors and tumor-draining lymph nodes. They also reported that butyrate reduced antigen-presenting cell (APC) activation and function and hence impaired the radiation-induced anti-tumor response [57] (Figure 2). Conversely, Then et al. [55] showed in mice that a soluble high-fiber diet delayed tumor growth following irradiation and was associated with a high abundance of *Bacteroides acidifaciens.* Using in vitro models of bladder cancer, this effect was suggested to be via an increase in SCFA production (Figure 2). While much of this pre-clinical evidence suggests that butyrate alone may not have a beneficial effect on the efficacy of radiotherapy-based treatment regimens, the clinical evidence described did show beneficial effects of butyrate. It is possible that the effects of butyrate alone are tempered when in combination with other SCFAs and other metabolites in a clinical population. Further metabolomic analyses are likely required to completely understand these relationships. 

## 4. SCFAs and Cancer Treatment Toxicities

**Figure 3:**
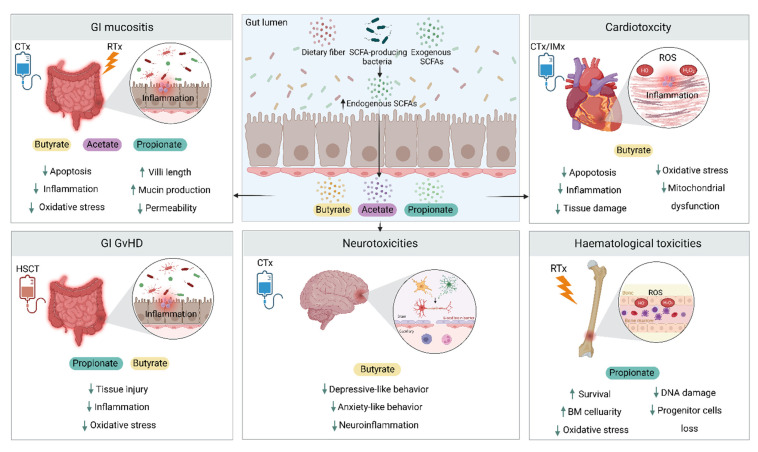
The role of SCFAs in cancer treatment toxicities based on current evidence. SCFAs can alleviate chemotherapy, radiotherapy, and HSCT-induced GI damage associated with mucositis and GvHD by reducing cell death, inflammatory and oxidative stress responses, and decreasing intestinal permeability by protecting villi length and enhancing mucin production. Butyrate protects against chemotherapy-related cardiac toxicities by reducing tissue injury, inflammation, oxidative stress, and mitochondrial dysfunction. Butyrate also has beneficial effects against chemotherapy-induced behavioral changes and neuroinflammation. Propionate protects against hematological toxicities by enhancing bone marrow cellularity and reducing DNA damage, oxidative stress, and progenitor cell death. SCFAs, short chain fatty acids; GI, gastrointestinal; CTx, chemotherapy; RTx, radiotherapy; IMx, immunotherapy; ROS, reactive oxygen species; GvHD, graft-versus-host disease; HSCT, hematopoietic stem cell transplantation.

Cancer therapies are associated with a wide range of adverse events, including gastrointestinal, cardiovascular, hematological, and psychoneurological toxicities (Table 2). These toxicities not only have significant short- and long-term impacts on patients’ quality of life but also negatively affect treatment efficacy and patient prognosis. The pathophysiology of these toxicities represents complex processes and involves the interaction between tissue injury, oxidative stress, and inflammation [65,66]. Given the protective, anti-inflammatory, and antioxidant properties of SCFAs [67], these metabolites have been found to attenuate the severity of these toxicities. This effect is mediated by attenuation of tissue damage, inflammatory responses, and or oxidative stress and improving intestinal integrity and reducing intestinal permeability (Figure 3). 

**Table 2:** Impact of SCFA-based interventions on cancer treatment toxicities.

Ref.	Subjects/Model	Treatment	Toxicity	Key Findings
da Silva Ferreira et al. [68]	3D intestinal organoids	Methotrexate +/− SCFAs	GI toxicity	Butyrate and propionate reduced the reduction in metabolic activity caused by methotrexate.
Ferreira et al. [69]	Mice	5-FU +/− SCFAs/ butyrate	GI toxicity	SCFA and Butyrate reduced or prevented the 5-FU-induced reduction in body weight and intestinal length. SCFA and Butyrate reduced tissue damage and mucosal ulceration in the small intestine. SCFA and Butyrate had no effect on inflammatory infiltrates but prevented 5-FU induced increase in EPO enzyme activity (a marker for eosinophil infiltration). Butyrate decreased intestinal permeability and ZO-1 expression associated with 5-FU.
Yue et al. [70]	THP-1 cells and Caco-2 cells Mice	5-FU +/− SCFAs/ *L. rhamnoides*	GI toxicity	SCFAs (in vitro): Suppressed ROS production Reduced expression of NLRP3 and proinflammatory cytokines Reduce autophagy markers. *Lactobacillus rhamnoides* (in vivo): Increased fecal SCFAs Increased serum IL-1β, IL-6 and IgA Decreased splenic NLRP3 and IL-17 Increased intestinal ZO-1 and occludin
Wang et al. [71]	Colon carcinoma-bearing mice	5-FU +/− Carboxymethylated pachyman (modified polysaccharide)	GI toxicity	Acetate, propionate, and butyrate reduced while isobutyrate and isovalerate increased following 5-FU. Carboxymethylated pachyman restored normal levels of SCFAs Intervention reduced 5-FU-induced intestinal tissue injury, apoptosis, and inflammation.
Panebianco et al. [46]	Pancreatic adenocarcinoma-bearing mice	Gemcitabine +/− butyrate	GI toxicity	Butyrate attenuated toxicity by protecting villi structure, increasing mucin production, and enrichment of anti-inflammatory SCFA-producing microbiota.
Guo et al. [72]	Mice	TBI (8 – 8.2 Gy) +/− SCFAs	Haemopoietic + GI toxicity	Radiation-resistant mice had higher concentrations of fecal total SCFA and propionate. Propionate enhanced the survival rate. Propionate increased bone marrow cellularity and splenic pulp recovery and reduced the radiation-induced loss of hematopoietic progenitor cells. Propionate increased crypt length and mucus thickness. SCFAs attenuated DNA damage and reactive oxygen species production in hematopoietic and gastrointestinal tissues
Huang et al. [73]	Mice	Doxorubicin +/− sodium butyrate	Cardiotoxicity	Doxorubicin reduced levels of fecal and serum butyrate Butyrate increased arginase-1 and CD206 levels and decreased cardiomyocyte apoptosis and myocardial enzymes. Butyrate promotes the polarization of colonic anti-inflammatory M2 macrophages.
Russo et al. [74]	Mice Cardiomyocytes/ endothelial cells	Doxorubicin +/−butyrate derivative (phenylalanine-butyramide (FBA))	Cardiotoxicity	FBA: Reduced Doxorubicin-induced left ventricle dilatation and volume Prevented fibrosis, apoptosis, and reduction in cardiomyocyte size Reduced expression of cardiac dysfunction and remodeling markers Reduced oxidative stress markers and prevented mitochondrial dysfunction Prevented cell damage and apoptosis.
Chen et al. [75]	Melanoma-bearing mice	PD-1/PD-L1 inhibitor +/− *P. loescheii/* butyrate	Cardiotoxicity	Lower fecal butyrate in cardiotoxicity model SCFA-producing bacteria (*P. loescheii*) or butyrate reduced myocardial apoptosis and serum myocardial enzymes. *P. loescheii* and butyrate downregulated proinflammatory factors in the colonic and cardiac tissues.
Mathewson et al. [76]	Mice	BMT +/− butyrate / butyrate-producing Clostridia strains	GI GVHD	BMT reduced intestinal butyrate and reduced histone acetylation in IECs. Butyrate restored histone acetylation, reduced apoptosis, and enhanced tight junction integrity. Butyrate + Clostridia strains attenuated GI GVHD severity.
Cristiano et al. [77]	Mice	Paclitaxel +/− sodium butyrate	GI + behavioral dysfunctions	Paclitaxel reduced intestinal barrier integrity, caused microbial dysbiosis, and decreased fecal butyrate. Oral butyrate attenuated disruption of intestinal barrier integrity and microbial dysbiosis. Oral butyrate attenuated depressive and anxiety-like behavior and neuroinflammation.

### 4.1. Gastrointestinal Toxicity

Due to the high sensitivity of gastrointestinal mucosa to cytotoxic agents, almost all cancer treatments cause some degree of mucosal injury along the gastrointestinal tract. This is observed in 80-100% of patients depending on treatment type and site, and dose [78]. Gastrointestinal mucositis (GIM), inflammation of the lower intestinal mucosa, is one of the most common and distressing adverse reactions of cancer therapies including chemotherapy, radiotherapy, and immunotherapy. GIM is characterized by initial DNA damage and production of reactive oxygen species (ROS), activation of inflammatory responses and production of pro-inflammatory mediators, tissue injury, and impairment of intestinal barrier functions [79]. As a result of these factors, gut microbial dysbiosis, an imbalance in the number and/or type of bacteria comprising the microbiota, is also observed [79]. Due to the close relationship between the types of bacteria in the microbiome and the SCFAs produced, this dysbiosis would therefore suggest changes in the amounts and types of SCFAs in GIM. As such, the gut microbiota and its metabolites could be a target for the management of GIM. 

SCFAs can influence the pathogenesis of intestinal injury by promoting crypt cell proliferation, modulation of immune responses, and maintenance of intestinal barrier integrity [80]. Therefore, the protective effect of SCFA against cancer treatment-induced intestinal injury has been an area of interest for decades. For instance, Ramos et al. showed in 1997 that the administration of SCFAs reduced histological damage and inflammation in the intestine of mice treated with the chemotherapeutic drug cytarabine [81]. Since then, several studies have investigated the protective effect of SCFA in both in vitro and in vivo GIM models of several chemotherapeutic agents as well as radiotherapy. For instance, in an in vitro study, SCFAs, mainly butyrate, attenuated methotrexate-induced toxicity in a 3D intestinal organoids model [68]. In mice treated with 5-FU, Ferreira et al. demonstrated that the administration of mixed SCFAs reduced intestinal injury; however, it did not impact intestinal permeability. Conversely, when butyrate was administrated alone, it resulted in a similar result with a reduction in intestinal permeability [69]. In another model of 5-FU-induced GI toxicity, treating human mononuclear macrophage (THP-1) and colorectal adenocarcinoma (Caco-2) cells with any of acetate, butyrate, and propionate, reduced cell death and suppressed the production of ROS and expression of proinflammatory mediators including NLRP3 inflammasome and cytokines in both cell lines compared to 5-FU without SCFAs. The study also reported a reduction in autophagy markers, LC3-II and Beclin-1, in THP-1 cells and increased expression of mucosal barrier markers, occludin, and MUC2, in Caco-2 cells. In the same study, the oral administration of SCFA- producing *Lactobacillus rhamnosus (L. rhamnosus)* in a mouse model of 5-FU induced GIM restored the reduction in fecal SCFAs and increased the expression of intestinal ZO-1 and occludin and serum IgA. *L. rhamnosus* also reduced IL-1β and IL-6 in serum and spleen, splenic NLRP3 inflammasome, and increased splenic anti-inflammatory IL-10 [70]. Furthermore, Gallotti et al. demonstrated that a high-fiber diet enriched with SCFA-producing *Bifidobacterium* and *Lactobacillus* was associated with improved intestinal histopathological changes and decreased intestinal permeability in mice model of irinotecan-induced mucositis. However, administering free acetate had no impact on both the histopathology and intestinal permeability, but it did reduce immune cell infiltration and attenuate inflammation in the same model. The lack of effect of free acetate on intestinal tissue injury or permeability could be due to the fact that acetate is mostly absorbed in the upper GI tract (esophagus, stomach, and small intestine) [82] and hence only a small fraction may reach the lower GI tract. This suggests that modulating the gut microbiota with diet, to enhance the production of endogenous SCFAs, may be more beneficial than orally administrating SCFAs, which could be rapidly metabolized [83]. Other agents that restore the normal levels of SCFAs have also been found associated with lower 5-FU GI toxicity. Wang et al. reported that the use of the polysaccharide, Carboxymethylated pachyman, in a colon tumor-bearing mouse model, reduced 5-FU-associated colon injury by reducing oxidative and inflammatory mediators. This agent also prevented gut microbial dysbiosis and the reduction in fecal SCFAs caused by 5-FU treatment [71]. SCFAs may also help protect against GI toxicity of gemcitabine, a standard treatment for pancreatic cancer. It has been shown that, in a pancreatic cancer mouse model treated with gemcitabine, butyrate supplements can reduce intestinal injury by preserving villi structure, increasing mucin production, enriching butyrate and propionate-producing bacteria, and suppressing pro-inflammatory microbes [46]. 

SCFAS may also play an important role in radiotherapy-induced GIM through the attenuation of inflammation [84]. Clinically, Ferreira et al. reported a significant reduction in butyrate levels during pelvic radiotherapy, and this was correlated to higher GI toxicities in patients with prostate cancer [85]. Further, the administration of SCFA- producing *Lachnospiraceae* strains or propionate protected against radiation and improved animal survival in a mouse model of total body irradiation. Propionate ameliorated GI injury by increasing crypt length and mucus thickness while both propionate and butyrate treatment reduced DNA damage markers in intestinal epithelial cells [72]. 

Another form of GI toxicity is radiotherapy-induced proctitis—inflammation of the rectal mucosa. This is a common complication in patients treated with radiotherapy for pelvic malignancies including rectal, bladder, gynecological, and prostate cancer [86]. Multiple studies have clinically investigated the effectiveness of butyrate in alleviating proctitis. This includes two small studies and one more recent large study. Vernia et al. evaluated the efficacy of topical butyrate in treating acute radiation proctitis in 20 patients receiving radiotherapy for pelvic malignancies and reported that butyrate led to the remission of proctitis symptoms [87]. In another small study that included 31 patients with prostate cancer, Hille et al. reported that sodium butyrate enema significantly reduced the incidence and severity of acute radiation proctitis but had no effect on late proctitis [88]. Conversely, a randomized placebo-controlled phase 2 trial, that included 166 patients with prostate cancer, found that the daily administration of increasing doses of sodium butyrate enemas had no significant impact on the incidence, severity, or duration of acute radiation proctitis [89]. As such, the current evidence does not support butyrate as an intervention for radiation proctitis [90]. Alternatively, Sasidharan et al. investigated whether oral resistant starch supplements could be used to prevent acute radiation proctitis in patients with cervical cancer treated with chemoradiotherapy. The study reported no difference between patients who received resistant starch and those who received digestible starch in terms of the severity of clinical and functional proctitis. The study also reported no difference in the levels of fecal SCFAs between groups [91]. The use of concurrent chemotherapy, which negatively impacts the gut microbiota composition and intestinal permeability, could limit the positive impact of resistant starch supplements and may explain why there was no change in SCFAs levels in the intervention group. This suggests that butyrate enema and resistant starch are not effective against radiation proctitis, and hence alternative SCFAs administration approaches need to be investigated.

Collectively, accumulating evidence supports the role of SCFAs in protecting against cancer treatment-related GI toxicities (Figure 3). Although the use of SCFAs orally [83] or as enemas [89] has shown limited success, the use of a fiber-rich diet to enhance the production of endogenous SCFAs offers a more effective approach [83]. Further research into the clinical implication of SCFAs and the best SCFA-based interventional strategy is warranted. 

### 4.2. Cardiotoxicity 

Cardiotoxicity is one of the life-threatening complications of cancer therapies including doxorubicin chemotherapy and programmed cell death-1 (PD-1) and programmed death-ligand 1 (PD-L1) inhibitor immunotherapies [92]. Generally, cardiotoxicity can manifest as myocarditis, cardiac fibrosis, cardiomyopathy /heart block, or heart failure [93,94]. Doxorubicin’s cardiotoxic effects are widely attributed to oxidative stress, which is characterized by increased accumulation of iron and the production of ROS in myocardiocytes. Increased levels of iron and ROS result in mitochondrial dysfunction and myocardial damage. This impacts cardiac contractile function and cardiac dilatation and may eventually lead to cardiac dysfunction [95,96]. Gut microbial dysbiosis and activation of immune response could contribute to doxorubicin-induced cardiotoxicity [73]. In terms of immunotherapy-associated cardiac toxicity, the proposed mechanism involves the activation of immune responses, increased expression of proinflammatory mediators, and increased infiltration of activated T-cell lymphocytes into the myocardium [97], which could also be altered by systemic SCFAs. 

Due to their antioxidant and anti-inflammatory properties, SCFAs could modulate chemotherapy and immunotherapy-related cardiac side effects; however, this only has been investigated in a few pre-clinical studies. Russo et al. demonstrated that the butyric acid derivative phenylalanine-butyramide (FBA) protects against changes in left ventricle dilatation and systolic and diastolic volume in mice treated with doxorubicin [74]. This effect was mediated by reducing cardiac fibrosis and apoptosis as well as reducing the levels of nitrosative (nitrotyrosine and nitric oxide synthase) and oxidative stress (hydrogen peroxide and mitochondrial superoxide dismutase) mediators, hence improving mitochondrial dysfunction. This study also reported a protective effect of FBA against cell damage and apoptosis in doxorubicin-treated cardiomyocytes and endothelial cells in vitro. More importantly, treating breast cancer cells with combined FBA and doxorubicin did not affect the anti-tumor activity of doxorubicin [74]. In another pre-clinical study, doxorubicin caused gut microbiota dysbiosis and reduced fecal and serum butyrate levels, and this was associated with cardiotoxicity. Furthermore, oral administration of butyrate reduced cardiomyocyte apoptosis and induced an anti-inflammatory effect by promoting the polarization of the anti-inflammatory M2 macrophages in the colon [73]. Similarly, Chen et al. demonstrated that the PD-1/ PD-L1 inhibitor-induced cardiotoxicity mouse model was associated with gut microbial dysbiosis characterized by a significant reduction in SCFA-producing *Prevotellaceae* and *Rikenellaceae* and lower production of butyrate. The oral administration of butyrate-producing *Prevotella loescheii* or butyrate itself attenuated cardiotoxicity by reducing the expression of pro-inflammatory cytokines IL-1β and TNF-α and M1 macrophages polarization in the colon resulting in lower inflammatory responses [75]. 

Together, current evidence suggests that microbiota-derived butyrate alleviates chemotherapy and immunotherapy cardiotoxic effects by decreasing oxidative stress, ROS production, and apoptosis in cardiac tissues as well as attenuating inflammatory responses (Figure 3). However, these have only been investigated in a few pre-clinical studies. Further research is required to validate these findings and to assess the effectiveness of butyrate treatments on cardiotoxicity clinically. 

### 4.3. Hematological Toxicities 

Several cancer treatments are associated with hematological complications including bone marrow suppression, and reduction in hematopoietic progenitor cells. This results in anemia, neutropenia, and a higher risk of infections [98,99]. Gut microbiota-derived SCFAs have been found to modulate hematopoiesis in the bone marrow to regulate inflammation in different body sites [100]. As such, SCFAs could play a protective role in cancer treatment-associated hematological toxicities. In mouse models of total body irradiation, a therapy used in combination with high-dose chemotherapy for hematopoietic stem cell transplant (HSCT) conditioning and associated with significant hematopoietic toxicity, survival was positively associated with levels of fecal total SCFAs and propionate. Further, the study showed that the oral administration of high-SCFA producer *Lachnospiraceae* strains or propionate supplements protected mice against radiation and improved survival. This was also associated with increased bone marrow cellularity and spleen pulps recovery. Further, propionate promoted hematopoiesis and reduced the loss of hematopoietic progenitor cells including megakaryocyte-erythroid progenitors and granulocyte-macrophage progenitors. Additionally, both butyrate and propionate were able to reduce DNA damage markers and levels of ROS in bone marrow-derived cells [72]. Finally, one study of patients with advanced esophageal cancer receiving neoadjuvant chemotherapy tested synbiotics as a supportive care strategy [101]. Participants receiving synbiotics had more positive outcomes than participants receiving antibiotics, with the occurrence of febrile neutropenia and the severity of diarrhea significantly inversely correlated with acetic acid concentration [101]. Together, preliminary evidence suggests that SCFAs, particularly propionate, could protect hematopoietic tissues against cytotoxic agents.

### 4.4. Graft Versus Host Disease 

HSCT prepared using donor stem cells (allogeneic/allo-HSCT) can cause life-threatening graft versus host disease (GvHD); a multisystem condition affecting several body organs including the gastrointestinal tract, skin, liver, and lung, manifesting acutely (aGvHD) and/or chronically (cGvHD) reaction [102]. GI injury is a major acute manifestation of GvHD characterized by diarrhea and abdominal pain. GI GvHD pathophysiology involves tissue injury and inflammation caused either by pre-HSCT conditioning regimens or the subsequent activation of donor APCs cells post-HSCT. This leads to the disruption of intestinal barrier integrity and tissue injury leading to enhanced production of proinflammatory cytokines including TNF-α and IL-1 [103]. 

Currently, there is growing evidence supporting a role of SCFA depletion in the development and severity of both aGvHD and cGvHD. It has been shown that allogeneic HSCT causes a significant reduction in SCFA-producing taxa and fecal levels of the three major SCFAs and this is associated with the severity of aGvHD [104]. Further, another study has shown that patients who did not develop acute GvHD had significantly higher levels of total fecal SCFA and propionate prior to HSCT [105]. This association between aGvHD development and lower levels of fecal SCFAs was also observed in pediatric patients undergoing allogeneic HSCT [106]. Additionally, lower levels of plasma propionate and butyrate were reported in allogeneic HCT recipients who developed cGVHD [107]. In contrast, butyrate levels in the stool collected at baseline or HSCT engraftment (two weeks post-HSCT) were not associated with GI aGvHD; however, those with lower pre-HSCT butyrate levels had a high risk of bloodstream infections [108]. In an experimental mouse model of allogeneic bone marrow transplant (BMT) with irradiation preconditioning, BMT was associated with a significant reduction in butyrate in intestinal tissues but not in serum or stool samples, and this was associated with a reduction in histone H4 acetylation in intestinal epithelial cells (IECs). The reduction in IECs butyrate was found to be caused by a reduction in butyrate uptake due to inflammation-induced downregulation of butyrate receptors and transporters. Conversely, the oral administration of butyrate or butyrate-producing bacteria restored histone acetylation and was associated with less weight loss and GvHD severity score, and increased survival. The study also suggested that the protective effect of butyrate was mediated by improving intestinal barrier integrity and reducing apoptosis [76]. In another study, both butyrate and propionate showed protection against GvHD, and this effect was mediated by the GPR43 signaling pathway [109]. 

Overall, similar to GIM, most of the present research supports a protective role for SCFAs in GI injury associated with GvHD (Figure 3). Again, further research into the best approach for intervention is warranted. 

### 4.5. Psychoneurological Toxicities 

Although a relatively strong body of evidence exists to support the role of the microbiota, and indeed SCFAs in modulating the toxicities described above, emerging data is now beginning to suggest a possible role in psychoneurological symptoms. This cluster of related symptoms includes cognitive impairments affecting memory, executive function, processing speeds, and learning [110,111,112], as well as psychological elements including depression/anxiety, fear of recurrence, and personality changes [113,114]. Although the current evidence is limited, this mechanism is supported by: i) changes in the gut microbiota that coincide with psychoneurological symptoms in people with cancer, and ii) direct involvement of SCFAs in other neurological conditions that share similar symptom profiles.

The first evidence of an association between gastrointestinal microbiota composition, post-chemotherapy, and fear of cancer recurrence was reported with lower microbial diversity, increased *Bacteroidetes,* and decreased *Firmicutes* at phylum level linked with symptom occurrence in survivors of breast cancer [115]. Additionally, a pilot study investigating the link between psychosocial factors and changes to gastrointestinal microbiota composition in 12 survivors of breast cancer, reported associations between fatigue and changes in SCFA-producing *Faecalibacterium* and *Prevotella* abundance as well as anxiety and changes in *Coprococcus* and *Bacteroides* abundance [116]. 

Mechanistically, SCFAs are thought to be critical mediators in gut–brain communication due to their effect on blood–brain barrier permeability, microglial activity, neuronal function, and neuroinflammation [19]; each of which has been reported after cancer therapy [117,118]. Clinically, decreases in fecal SCFA concentrations have been associated with the presence of both major depressive disorder and Alzheimer’s disease (AD) [119,120]. Additionally, SCFA concentrations were closely linked to the progression of AD with levels decreasing progressively across control, mild cognitive impairment, and AD patient groups, following the deterioration seen in cognitive function [120]. Pre-clinical studies have also demonstrated the ability of SCFAs to mitigate symptoms of cognitive impairment in mouse models of AD, as well as cognitive impairment induced by a variety of other factors including radiation, isoflurane, and scopolamine [121,122,123,124]. Similar evidence has also been generated in Parkinson’s disease [125].

In the context of cancer therapies, only one pre-clinical study has directly investigated the impact of SCFAs on chemotherapy-associated behavioral dysfunctions. Cristiano et al. demonstrated that treating mice with the chemotherapeutic agent paclitaxel caused gut microbial dysbiosis, impaired intestinal barrier integrity, reduced fecal butyrate levels, and increased systemic inflammation. This was also associated with enhanced anxiety and depression-like behaviors as well as neuroinflammation. Conversely, the authors showed that the administration of oral butyrate restored the reduction in fecal butyrate levels and was able to minimize the alterations in the intestinal environment and improve behavioral changes and neuroinflammation [77]. Considering this, along with the mounting evidence implicating the microbiota-gut–brain axis in the development of the psychoneurological complications of chemotherapy treatment, SCFAs present as a feasible therapeutic target warranting further investigation in the context of neurotoxicity associated with cancer treatments.

## 5. Targeting SCFA to Improve Cancer Treatment Outcomes

Overall, current evidence suggests that SCFAs may provide a potential non-invasive target to enhance the efficacy and alleviate the toxicities of cancer therapies. This can be achieved through oral administration of SCFAs, the consumption of a fiber-rich diet to enrich indigenous SCFA-producing gut microbiota, or through the ingestion of SCFA-producing microorganisms. However, some evidence has shown that oral administration of SCFA has a limited impact due to rapid metabolism and absorption [51,83]. While free SCFAs can be used to increase the systemic levels of SCFAs, the consumption of dietary fiber or SCFAs-producing bacteria offer more effective tools to enhance the production of endogenous SCFAs. For example, the ingestion of SCFA-producing bacteria like *C. butyricum* has shown some positive impact in improving immunotherapy efficacy [53,54,126]. The use of chemically modified SCFAs with a more potent activity also has been explored. For instance, the use of the butyrate derivative, phenylalanine-butyramide (FBA), which has comparable chemical properties to butyrate but is more potent and palatable, could be used as an alternative to butyrate administration [74,127]. 

Alternative indirect methods of altering gut microbiota composition, and therefore SCFA-producing bacteria, might include the use of probiotics, prebiotics, or fecal microbiota transplant, all of which have been tested with varying success in cancer settings [2,128,129]. However, as more information comes to light regarding the actions of specific SCFAs in efficacy and toxicity, these methods may need to be refined to produce a specific, beneficial response. More precise methods to directly alter specific SCFA effects on a variety of cell types may be via activating particular SCFA receptors [130]. This has been previously shown by Singh et al, who showed that directly activating the butyrate receptor Gpr109a suppressed colonic inflammation and carcinogenesis [131]. These methods are still in their infancy in clinical practice. In developing methods of using SCFAs to improve efficacy and toxicity, awareness would need to be had around how these treatments would work for different populations of people, with different microbiota compositions, as well as different treatment regimens. In addition, balancing effects on efficacy and toxicity concurrently would need to be managed [132]. 

This paper has focused on SCFAs, as they are likely the most widely understood and studied bacterial metabolites, however other key classes of metabolites may also be useful research targets in the future for their role in enhancing cancer treatment efficacy and toxicity. For instance, inosine, tryptophan metabolites (Indole-3carboxaldehyde), and secondary bile acids have been found to improve immunotherapy outcomes [133,134]. Further, these metabolites may provide protection against therapy-related toxicities [72,135]. Additionally, microbiota-derived L-Histidine and imidazole propionate have shown a protective effect against radiation-induced cardiopulmonary toxicities in mice. A study showed that chest irradiation was associated with reduced fecal L-Histidine, while fecal microbiota transplant and L-Histidine and imidazole propionate oral supplement helped reduce lung and heart injuries following irradiation. Further analysis showed imidazole propionate can decrease pro-inflammatory mediators, inhibit inflammation-induced cell death, and prompt cellular proliferation [135].

We suggest that assessing microbiota composition alone is no longer sufficiently informative to properly understand the role of the microbiota in cancer treatment efficacy and toxicity, and untargeted metabolomic analyses (including SCFAs, indole-3carboxaldehyde, and secondary bile acids) will add an additional layer of actionable information. 

## 6. Current Limitations and Future Considerations 

Results from current studies suggest that the SCFAs based interventions could be used to promote cancer treatment efficacy and alleviate their toxicities. However, none of these interventions has been implemented clinically due to the limitations of the current evidence. For both treatment efficacy and toxicity, most of the present studies have investigated SCFAs impacts in pre-clinical settings with the majority of these studies focusing on chemotherapy and only a few studies on immunotherapy and radiotherapy. For the few clinical studies that have been conducted, they mainly aimed to evaluate the association between SCFAs levels and treatment outcomes and not to investigate SCFAs as an intervention. Further, some of these studies have often not been completed in a linear fashion, with clinical studies occurring before pre-clinical studies. Additionally, there is still a lack of understanding of the most efficient SCFA-based intervention strategy for each treatment outcome. Moreover, different studies have used different methodological approaches resulting in inconsistent outcomes. All these limitations need to be addressed in future studies. 

The use of different methodologies could have critical implications on studies outcomes, and this also may explain some of the inconsistencies observed across the different studies described here. As such, different factors related to study design including the type of models (in vitro cell lines or in vivo models), type of SCFAs, type of dietary fibers, and SCFAs source or administration method (free SCFAs, SCFAs complexes, or microbiota-produced SCFAs) should be considered when designing future SCFAs studies depending on the target sites and the intended impact of SCFAs interventions (local or systemic). Firstly, the use of in vitro models can only be used to assess the local effects of SCFAs. For instance, treating cell lines with butyrate will only reflect its local effects and is unlikely to reflect its effects in tumors at distant sites because most butyrate is used by colonic cells and only a small fraction (~2%) can reach the systemic circulation [18]. As such, to assess the systemic effects of butyrate or other SCFAs, in vivo pre-clinical and clinical studies are needed. Further, direct administration of SCFAs or methods to promote SCFA production are important determinants of their local and systemic effects. To achieve local effects in the colon, dietary fiber or SCFAs-producing probiotics could be used to enhance the production of endogenous SCFAs. Conversely, for direct SCFA action in tumors in the upper GI, such as in gastric cancer, or in other distant tumors, administration of free SCFAs would be potentially more effective. This is because oral SCFAs could be absorbed in the upper GI tract and reach the systemic circulation, inducing a beneficial effect on local upper GI tract epithelium and distant body sites [136]. However, the effects of gut microbiota-derived SCFAs related to colonic anti-inflammatory pathways that may also affect distal sites should not be neglected. It is also worth mentioning that while the use of fermentable fibers can increase the levels of systemic SCFAs [137]; the interindividual variations in SCFA production may impose a limitation on this approach. Another factor that may impact study outcomes is the type of dietary fiber used. In studies using this method, the type of each dietary fiber should be carefully considered as different types of fibers can promote the growth of different bacterial types, and therefore the production of different types of SCFAs [138]. 

Generally, in future studies, it would be ideal to complete high throughput-screening of a wide range of SCFA-based products in cell culture models to identify potential leads, before using animal models (ideally with a humanized microbiota) to assess effects on toxicity and efficacy in a range of tumor and treatment settings. Finally, these results could be tested in clinical studies. The most appropriate experimental settings, type of SCFAs or SCFAs-based products as well as administration method should be taken into account for both pre-clinical and clinical studies.

## 7. Conclusions

Microbiota-derived SCFA are important regulators of intestinal cell function, as well as local and systemic immune and inflammatory responses. Given this, they have been found to influence cancer treatment efficacy, through modulation of immune functions, and toxicity, through their protective epithelial functions and anti-inflammatory effects. Determining the best strategy to target SCFAs and their broad effects to improve cancer treatment outcomes will be important.
